# Clinical Features and Gene Mutation Analysis of Congenital Thrombotic Thrombocytopenic Purpura in Neonates

**DOI:** 10.3389/fped.2020.546248

**Published:** 2020-09-22

**Authors:** Jiali Wang, Li Zhao

**Affiliations:** Department of Neonatology, Children's Hospital of Nanjing Medical University, Nanjing, China

**Keywords:** congenital thrombotic thrombocytopenic purpura, ADAMTS 13, gene mutation, neonates, congenital disease, genetic testing, family history

## Abstract

Congenital thrombotic thrombocytopenic purpura (TTP) is a rare hereditary disease with a high mortality rate; however, improved patient survival is possible with prompt diagnosis and treatment. The clinical features and mutation sites of a disintegrin and metalloproteinase with thrombospondin motifs *13* (*ADAMTS13*) in congenital TTP were analyzed in a neonate with suspected congenital TTP. High-throughput sequencing, polymerase chain reaction, and Sanger sequencing were utilized for screening of genes related to thrombocytopenic diseases and *ADAMTS13* gene mutation testing on blood samples from the neonate and the parents. Domestic and foreign literature reporting the clinical features and variants of *ADAMTS13* in neonates with congenital TTP were retrieved, compared, and analyzed. The patient in this case was a girl who had been born for 1 h and admitted to the hospital due to “dyspnea for 1 h.” Routine blood tests on admission revealed profound thrombocytopenia. She quickly developed symptoms of systemic hemorrhage and eventually died. The neonate had two older sisters who had died of idiopathic thrombocytopenia and hemorrhage within 24 h of birth. Genetic testing showed that the neonate harbored a compound heterozygous mutation in *ADAMTS13*, c.1187G>A/c.1595G>T, which is a novel variant. Of the 12 cases (1 case in China and 11 cases in other countries) of congenital TTP in neonates that have been reported globally, *ADAMTS13* mutation analysis was only performed in eight neonates. Common clinical manifestations included dyspnea of unknown etiology, bruising, jaundice, hemorrhage, and thrombocytopenia. Hence, the current case contributes to our understanding of the clinical manifestations and types of variants in neonates with congenital TTP. Our results demonstrate the efficacy of high-throughput sequencing technology in genetic testing of neonates suspected with congenital TTP and have revealed a novel compound missense mutation in *ADAMTS13* that has not been reported in China or elsewhere.

## Introduction

Congenital thrombotic thrombocytopenic purpura (TTP) is a rare hereditary disease characterized by periodic episodes of microangiopathy, hemolytic anemia, thrombocytopenia, and organ damage of variable severity. These symptoms may occur during the neonatal period, but their first appearance may also occur after adulthood. If not treated promptly, onset of congenital TTP has a high mortality rate. However, most patients can survive if timely diagnosis and treatment are provided (1). The relevant TTP registry showed that the annual incidence of congenital TTP is (1-3)/1,000,000 (2); however, the pathogenesis of TTP remains unclear. Currently, the prevailing opinion is that the cause of TTP is multifactorial, including extensive injury of the vascular endothelium, reduced prostacyclin concentration in blood, and the formation of von Willebrand factor (VWF) multimers due to impaired cleavage of ultra-large von Willebrand factor (UL-VWF), in addition to the damaging effects of autoantibodies and oxidants that promote platelet activation. In recent years, the discovery of UL-VWF multimers, purification of VWF-cleaving protease (VWF-CP), and cloning of *ADAMTS13* have led to the belief that a deficiency of the ADAMTS13 enzyme is the major mechanism underpinning TTP pathogenesis (3). Additionally, thrombin-sensitive protein 1 (TSP1), abnormal complement regulation, anti-endothelial cell antibody (AE-CA), and excessive release of VWF multimers have been associated with the onset of TTP (4). Under physiological conditions, the ADAMTS13 enzyme in the plasma specifically cleaves VWF multimers into small molecular fragments, thereby regulating normal coagulation function. When ADAMTS13 enzymes are deficient, such as due to the presence of *ADAMTS13* mutations or autoantibodies against the ADAMTS13 enzyme, the VWF multimers cannot be cleaved normally. This leads to a large amount of UL-VWF in blood circulation, resulting in extensive microvascular thrombosis and damage to multiple organs, thereby triggering the onset of TTP. A significant reduction in ADAMTS13 enzyme activity and the negativity of antibody against ADAMTS13 enzyme detected through laboratory testing are the key indicators of TTP diagnosis. Meanwhile, the combination of high-throughput sequencing, polymerase chain reaction (PCR), and Sanger sequencing can be used for prenatal screening and diagnosis in individuals with a family history of TTP. In this study, genetic screening was performed in a neonate with a suspected diagnosis of congenital TTP. Pedigree verification was conducted for the mutant gene identified, and the congenital TTP diagnosis was confirmed. Finally, the clinical features and genetic variants of congenital TTP in neonates from domestic and foreign reports were analyzed.

## Materials and Methods

### General Information

Our patient's father signed a written consent for publication of her clinical information and her pictures according to Children's Hospital of Nanjing Medical University's internal policies and statutes. The patient was a girl who had been born for 1 h. She was the third child carried and delivered by the mother. Her gestational age was 39^+3^ weeks. The mother had chronic hypertension with pre-eclampsia, and the patient was delivered vaginally. Her birth weight was 3,600 g, and her Apgar scores at 1 and 5 min were 8 and 9, respectively. The mother had been pregnant and had live-born baby girls twice before; however, both girls had died of jaundice and hemorrhage within 24 h of birth. The neonatal patient in the present case was admitted to the hospital due to dyspnea. Hemorrhage sites appeared throughout the body immediately after admission, and the number of sites gradually increased. Mechanical ventilation was provided to alleviate uncontrolled hemorrhage at puncture sites and symptoms of pulmonary hemorrhage. Prothrombin complex was administered for hemostasis, and platelet transfusion was performed. However, the symptoms of the patient did not improve. The parents gave up treatment 6 h after admission and the patient died. The first child was born and died in the obstetrics and gynecology department of a different hospital; however, details of laboratory test results and treatments provided were not available. The second child was admitted to our hospital after birth with jaundice. Upon admission, the patient exhibited symptoms such as oliguria, edema, systemic hemorrhage, uncontrolled hemorrhage at puncture sites, and progressive thrombocytopenia. Anti-infection and hemostatic treatments were given. The neonatal patient died 9 h after admission. The laboratory test results for the second and third births are shown in Table 1. None of the three neonatal patients were tested for ADAMTSl3 enzyme activity and antibodies.

**Table 1:** Laboratory test results for two of the live-born children of the neonatal patient's mother.

	**Hemoglobin (g/L)** **(140–180)**		**Platelets (×10**^****9****^**/L)** **(100–300)**		**MCV (fL)** **(80–100)**		**Lactate dehydrogenase (U/L) (185–407)**	**Bilirubin (μmol/L) (3.4–17.1)**	**International Normalized Ratio (INR) (0.82–1.15)**
	**At admission**	**Before death**	**At admission**	**Before death**	**At admission**	**Before death**			
2nd birth	65	30	146	28	97.7	104.2	2,897	433.78	2.38
3rd birth	163	73	20	18	112.6	92.3	1,368	153	1.37

### Screening for Inherited Metabolic Diseases

Peripheral blood (~2 ml) was drawn from the neonatal patient 5 h after admission and placed in a tube containing ethylenediaminetetraacetic acid (EDTA) as an anticoagulant. The sample was sent to the Beijing Full Spectrum Medical Laboratory for testing. Whole exome sequencing was performed using the xGen Exome Research Panel v1.0 from Integrated DNA Technologies (IDT) and sequencing was completed using the Illumina NovaSeq 6000 series sequencing system. The coverage of the target sequences was maintained >99%. Screening was conducted in a cloud-based platform, for precision diagnosis of thrombocytopenic diseases that integrated molecular biology annotations and analyses of biology, genetics, and clinical features. In addition, the pathogenic variant database, normal human genome database, and database containing the clinical features of 4,000 known genetic diseases were queried. Using the algorithms for genetic data analysis, several hundred thousands of genetic variants were classified. Variant classification was performed using the 3-tier classification system and the American College of Medical Genetics and Genomics (ACMG) variant classification system. PCR amplification of the target sequences was performed, and the sequences were validated through Sanger sequencing using the ABI3730 sequencer. Validation results were obtained using a sequence analysis software. Genetic testing results are shown in Table 2.

**Table 2:** Data on genetic variants.

**Gene**	**Serial number**	**Chromosome location**	**Nucleic acid changes**	**Amino acid changes**	**RS number**	**MAF**	**ACMG pathogenic variant class**	**Proband**	**Father**	**Mother**	**OMIM number of related diseases, mode of inheritance**
*ADAMTS13*	1	chr9:13 6303376	c.1595(exon 14)G>T	p.C532F (p.Cys532Phe)	None	Not recorded	Uncertain	Heterozygous 29/55	Wild type 0/54	Hybrid 30/61	TTP (OMIM: 274150), AR
	2	chr9:13 6298592	c.1187(exon 10)G>A	p.C396Y (p.Cys396Tyr)	None	Not recorded	Uncertain	Heterozygous 30/54	Heterozygous 49/102	Wild type 0/46	

### Literature Search

The keywords for literature search were “thrombotic thrombocytopenic purpura” and “Upshaw-Schulman syndrome.” Literature were retrieved from China National Knowledge Infrastructure, Wanfang Data, and PubMed using neonatal patients of ≤28-day-old as a criterion to obtain data and case reports of neonates with congenital TTP.

## Results

### Analysis of Clinical Features

Literature search revealed 12 cases of congenital TTP in neonates reported worldwide. The clinical features and prognoses of the cases are described in Table 3.

**Table 3:** Clinical features and prognoses of 12 neonates with congenital TTP.

**Case number**	**Sex**	**Time of onset**	**Jaundice**	**Hematuria**	**Anemia**	**Thrombocytopenia**	**Fever**	**Seizure**	**Abnormal renal function**	**ADAMTS13 activity**	**Family history**	**Outcome**	**References**
Case 1	Girl	40 h after birth	+	+	+	+	+	–	+	<1%	+	Deceased	(5)
Case 2	Boy	1 day after birth	+	+	+	+	–	–	–	5%	–	Survived	(6)
Case 3	Boy	3 days after birth	+	–	+	+	+	–	–	4%	+	Survived	(6)
Case 4	Girl	6 h after birth	+	+	+	+	–	+	–	10%	–	Survived	(7)
Case 5	Boy	11 h after birth	+	–	+	+	–	–	–	<0.5	–	Survived	(8)
Case 6	Girl	1 day after birth	+	–	–	+	–	–	+	0.5%	–	Survived	(8)
Case 7	Boy	4 h after birth	+	+	+	+	–	–	+	5%	–	Survived	(9)
Case 8	Boy	6 h after birth	+	–	+	+	–	–	+	<5%	–	Survived	(10)
Case 9	Girl	4 h after birth	+	+	+	+	–	–	+	10%	+	Survived	(11)
Case 10	Girl	1 day after birth	+	–	+	+	–	–	–	0.5%	–	Survived	(12)
Case 11	Boy	Not specified	+	–	+	+	–	–	–	–	–	Deceased	(13)
Case 12	Boy	27 days after birth	+	–	+	+	–	+	+	<10&	–	Deceased	(14)

### DNA Sequencing Results

**Figure 1:**
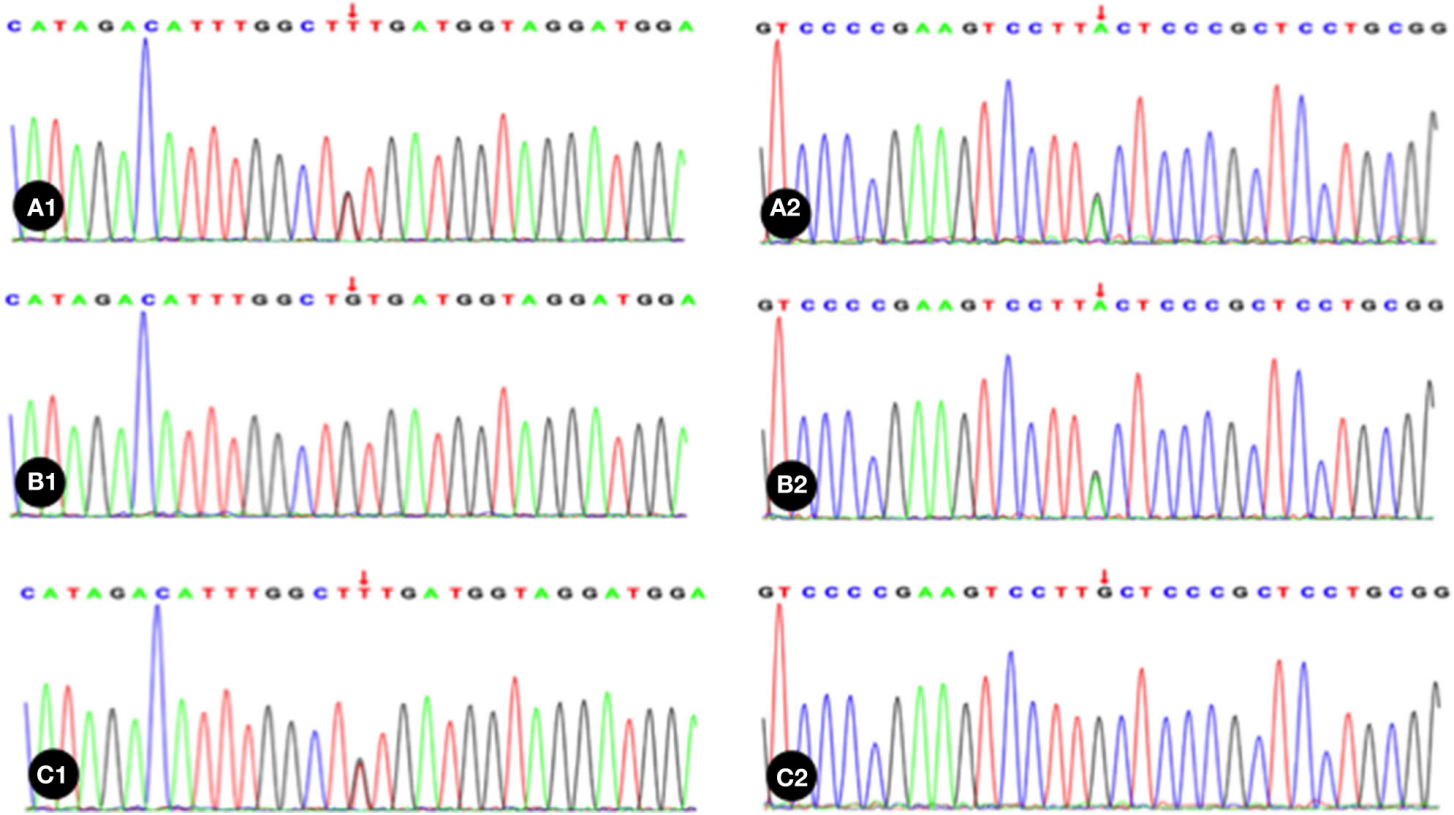
The neonatal patient harbored a mutation of c.1595G>T in 1 allele; B1, Absence of mutation in the patient's father; C1, The patient's mother harbored a heterozygous mutation of c.1187G>A; A2, The neonatal patient harbored a mutation of c.1187G>A in 1 allele; B2, The patient's father harbored a heterozygous mutation of c.1187G>A; C2, Absence of mutation in the patient's mother. Red arrow indicates the nucleotide position of the mutation.

Trio whole exome sequencing was performed in the Illumina NovaSeq 6000 series sequencing system. The exonic regions of about 20,000 genes in the human genome were sequenced. The coverage of the target sequences was no <99%. In the sample of the present case, *ADAMTS13* exhibited a c.1187G>A (p.C396Y) missense mutation in exon 10 and a c.1595G>T (p.C532F) missense mutation in exon 14. A search in the Human Gene Mutation Database (HGMD) did not reveal any published report on these 2 variants, suggesting that the mutations in these two locations are novel variants of *ADAMTS13*. Pedigree verification using PCR amplification and Sanger sequencing showed that the father of the neonatal patient harbored the c.1187G>A (p.C396Y) mutation, and the mother harbored the c.1595G>T (p.C532F) mutation (Figure 1). Hence, the patient's family met the criteria for the pathogenesis of autosomal recessive (AR) inheritance.

### *ADAMTS13* Mutation Analysis

Among the 12 cases reported thus far (5–14), gene mutation analysis was performed in eight cases (Table 4), one of which the mutation was not specified. The neonatal patient in case 1 only harbored one mutation, whereas the patients in the remaining six cases all harbored a compound heterozygous mutation; missense mutations and deletions in cases 3 and 5, and an insertion in case 7. The older brother of the patient in case 3 harbored the same gene mutation, but neonatal onset of the disease did not occur. The parents and two older brothers of the patient in case 5 were heterozygous carriers harboring one of the two mutations. In case 6, each parent harbored a mutant gene. Family members of the neonatal patients in the remaining three cases did not undergo genetic testing.

**Table 4:** Mutation analysis of the *ADAMTS13* in neonatal patients with congenital TTP.

**Case number**	**Genetic variant**	**References**
Case 1	c.2203(exon18)G>T	(5)
Case 2	c.2104(exon17)G>C; c.2281(exon19)G>A	(6)
Case 3	delEx15+16; c.3655C>T	(6)
Case 4	c.415-10G>A; c.824+13C>T; c.2017A>T	(7)
Case 5	c.1192(exon10) C>T; c.2167(exon18) C>A	(8)
Case 6	c.1345(exon 12)C>T; c.4119(exon 29)del	(8)
Case 7	c.3100(exon 24)A>T; c.4143(exon 29)ins A	(12)

## Discussion

The typical clinical manifestations of congenital TTP include five signs and symptoms: fever, microangiopathic hemolytic anemia, thrombocytopenia, renal dysfunction, and neurological symptoms. The neonate with congenital TTP in the present case and those in literature reports all exhibited severe signs of thrombocytopenia, jaundice and hemorrhage; however, none of the patients exhibited all five signs and symptoms, suggesting that congenital TTP lacked specific clinical features in neonates, and could be easily misdiagnosed as other conditions, such as immune thrombocytopenic purpura (ITP), Evans syndrome, hemolytic–uremic syndrome, or severe infection. Determination of ADAMTSl3 enzyme activity was conducted in 11 neonatal patients reported in the literature after the onset of disease, and the enzyme activity was <10% in all cases. Nine neonatal patients were treated with plasma exchange. They received regular fresh frozen plasma transfusions and achieved long-term survival. Of the 11 cases, only 2 deaths occurred. In one case, ADAMTSl3 enzyme activity was not determined. The patient died 34 h after birth, and postmortem genetic testing confirmed a diagnosis of congenital TTP. For the neonatal patient in the present case and her two sisters, ADAMTS13 enzyme activity and antibody testing were not performed immediately due to a lack of congenital TTP diagnosis confirmation. Thus, appropriate treatments, such as fresh frozen plasma transfusion were not provided, and the patients eventually died. Their diagnoses of congenital TTP were only confirmed via postmortem genetic testing. The results from our analysis of these cases suggest that a significant reduction in ADAMTSl3 enzyme activity is a key indicator of a congenital TTP diagnosis (15), such that a lower enzyme activity may correspond to an earlier onset in neonates (16, 17). Genetic testing can be used as an adjunct diagnostic tool. In addition, considering that the mother of the neonate in the present case had previously given birth to two live-born baby girls, both of whom died of jaundice and hemorrhage within 24 h of birth, the present case had a clear family history. Of the 12 neonates with congenital TTP reported in literature, three had a similar family history. Therefore, neonates exhibiting thrombocytopenia, severe hemorrhagic tendency, and jaundice of unknown etiology should raise suspicion of congenital TTP. In addition, early diagnosis and treatment should be performed to avoid adverse consequences. In inflammation, ADAMTS13 activity can also be reduced, but no more than 10%. Under certain circumstances, severe ADAMTSl3 enzyme deficiency could be masked by transfusions of any plasma-containing blood products (including plasma, red blood cell, or platelet transfusions). The testing of ADAMTS13 after plasma transfusion does not have any sense. Therefore, genetic testing may facilitate the diagnosis of congenital TTP (17). Furthermore, the PLASMIC score can be used as a rapid assessment tool for TTP (18). The PLASMIC scores assigned to the second and third births of the mother of the patient in the current case were 5 and 7, respectively.

In the present case, comprehensive genetic screening of genes related to thrombocytopenic diseases via high-throughput sequencing showed that *ADAMTS13* had a compound heterozygous mutation of c.1187 (exon10) G>A (p.C396Y)/c.1595G>T (p.C532F). Pedigree verification of these variants confirmed an autosomal recessive inheritance pattern. According to the ACMG guidelines, these two mutations are classified as variants of uncertain significance (VUS), indicating that they have not been reported in genomic databases or in literature. Taken together, the clinical features, PLASMIC score, family history, and the presence of *ADAMTS13* variants, strongly suggest congenital TTP in the present case. Additionally, the gene variant identified is a novel *ADAMTS13* variant.

The treatment for Upshaw-Schulman syndrome (USS) is relatively simple, which is the supplementation of the deficient ADAMTS13 enzyme. Currently, since no purified ADAMTS13 enzyme preparation exists, ADAMTS13 enzyme in patients may be supplemented with fresh frozen plasma. Intermediate-purity factor VIII concentrate or recombinant ADAMTS13 concentrate is potentially safer and more effective treatments (19). The use of recombinant ADAMTS13 concentrate is currently under investigations in clinical trials. The half-life of ADAMTS13 enzyme is 2–3 days, suggesting that the clinical effect of plasma or factor VIII concentrate transfusion can be maintained for 10–20 days. All neonatal patients who were transfused with fresh frozen plasma after a diagnosis had been confirmed, irrespective of on-demand or prophylactic treatments, achieved a favorable state of remission. Of the 12 cases reported in the literature, only 3 died. The remaining nine cases were treated with timely plasma exchange following a confirmed diagnosis. All patients displayed good prognosis following fresh frozen plasma transfusion. Long-term follow-ups also showed that all neonatal patients who received regular fresh frozen plasma transfusions achieved long-term remission. Prophylactic treatment is recommended for those with severe episodes combined with ischemic injury to organs or those with chronic forms of the disease. Transfusion should be given once every 2–4 weeks to maintain a safe platelet level and prevent clinical onset of disease. However, there is no consensus whether plasma therapy should be intensified to cover the most frequent triggering factors of TTP bouts in childhood, such as infections and vaccinations (20). In conclusion, congenital TTP is an inherited autosomal recessive disorder that is often mistaken for other conditions with immune-related cytopenias, such as ITP. Although the incidence is extremely low, neonatal patients who do not receive timely and accurate diagnosis and treatment will suffer severe adverse outcomes. In addition, accurate identification of congenital TTP relies on family history for initial diagnosis, ADAMTSl3 enzyme activity testing to confirm diagnosis, and genetic testing to facilitate diagnosis. The initiation of effective replacement therapy following a confirmed diagnosis can prevent relapse and improve the quality of life of neonatal patients with TTP.

## Data Availability Statement

All datasets generated for this study are included in the article/supplementary material.

## Ethics Statement

Written informed consent was obtained from the individual(s), and minor(s)' legal guardian/next of kin, for the publication of any potentially identifiable images or data included in this article.

## Author Contributions

JW conceptualized and designed the case report and reviewed and revised the manuscript. LZ developed initial draft, reviewed and revised the manuscript, and approved the final manuscript as submitted. All authors contributed to the article and approved the submitted version.

## Conflict of Interest

The authors declare that the research was conducted in the absence of any commercial or financial relationships that could be construed as a potential conflict of interest.
